# Weight Loss and Malnutrition in Patients with Parkinson's Disease: Current Knowledge and Future Prospects

**DOI:** 10.3389/fnagi.2018.00001

**Published:** 2018-01-19

**Authors:** Kai Ma, Nian Xiong, Yan Shen, Chao Han, Ling Liu, Guoxin Zhang, Luxi Wang, Shiyi Guo, Xingfang Guo, Yun Xia, Fang Wan, Jinsha Huang, Zhicheng Lin, Tao Wang

**Affiliations:** ^1^Department of Neurology, Union Hospital, Tongji Medical College, Huazhong University of Science and Technology, Wuhan, China; ^2^Department of Neurology, Anhui Provincial Hospital, The First Affiliated Hospital of University of Science and Technology of China, Hefei, China; ^3^Department of Psychiatry, Harvard Medical School, Division of Basic Neuroscience, and Mailman Neuroscience Research Center, McLean Hospital, Belmont, MA, United States

**Keywords:** weight loss, malnutrition, Parkinson's disease, energy homeostasis, weight evaluation, weight management

## Abstract

Parkinson's Disease (PD) is currently considered a systemic neurodegenerative disease manifested with not only motor but also non-motor symptoms. In particular, weight loss and malnutrition, a set of frequently neglected non-motor symptoms, are indeed negatively associated with the life quality of PD patients. Moreover, comorbidity of weight loss and malnutrition may impact disease progression, giving rise to dyskinesia, cognitive decline and orthostatic hypotension, and even resulting in disability and mortality. Nevertheless, the underlying mechanism of weight loss and malnutrition in PD remains obscure and possibly involving multitudinous, exogenous or endogenous, factors. What is more, there still does not exist any weight loss and malnutrition appraision standards and management strategies. Given this, here in this review, we elaborate the weight loss and malnutrition study status in PD and summarize potential determinants and mechanisms as well. In conclusion, we present current knowledge and future prospects of weight loss and malnutrition in the context of PD, aiming to appeal clinicians and researchers to pay a closer attention to this phenomena and enable better management and therapeutic strategies in future clinical practice.

## Introduction

Parkinson's Disease (PD), a progressive neurodegenerative disorder secondly only to Alzheimer's Disease (AD), is characterized by progressive dopaminergic neurons loss and Lewy body formation in the nigrostriatal system (Samii et al., 2004). Apart from nigrostriatal system, other nuclei such as locus ceruleus, brain stem reticular nuclei, dorsal motor nucleus of the vagus nerve, basal nucleus of Meynert, amygdala, and the CA2 area of the hippocampus (Chaudhuri et al., 2006) are also affected. As a matter of fact, PD is indeed a systemic disease manifesting with cardinal motor symptoms (bradykinesia, resting tremor, rigidity, and postural instability) as well as a series of non-motor symptoms, involving psychiatric symptoms, autonomic failure, gastrointestinal problems, sensory impairment, circadian rhythm disorder (Chaudhuri and Schapira, 2009) etc. Among these symptoms, weight loss is commonly documented, but neglected frequently and still remaining elusive up to now (Durrieu et al., 1992; Markus et al., 1993; Chen et al., 2003; Bachmann and Trenkwalder, 2006; Kashihara, 2006; Uc et al., 2006; Aziz et al., 2008; Kistner et al., 2014). Weight loss, primarily due to fat rather than muscle loss (Markus et al., 1993), appears to occur several years before the diagnosis and tends to continue during the disease process (Chen et al., 2003). In comparison with male subjects (−4.3%), female sufferers exhibit more significant weight loss (−8.5%) (Durrieu et al., 1992). Nevertheless, PD patients with normal weight and even overweight have been reported as well, especially in the region where overweight is prevalent (Barichella et al., 2003a; Morales-Briceno et al., 2012).

Occurrence of weight loss in PD has been associated with malnutrition and ensuing clinical problems such as falls, bone fracture (Fink et al., 2005; Genever et al., 2005; Schneider et al., 2008), infections (Wehren et al., 2003; Guttman et al., 2004) and worsening quality of life (Wills et al., 2016). In addition, weight loss and malnutrition complicates the course of PD inducing cognitive decline (Kim et al., 2012), orthostatic hypotension (Nakamura et al., 2017) and dyskinesia, resulting from intake of a greater cumulative levodopa dosage per kilogram of body weight (Zappia et al., 2002; Bachmann and Trenkwalder, 2006; Sharma et al., 2006).

A wealth of studies report weight loss may precede the diagnosis of PD by years and are associated with disease severity as well as duration (Lorefalt et al., 2004), indicating that a low body mass index (BMI) might be associated with PD-related pathologies. In addition, PD-related motor and no-motor symptoms may influence BMI as well. Whether weight loss is etiologically linked to PD, or is a consequence of the disease processes itself still remain obscure. For the time being, as revealed in other neurodegenerative diseases such as AD, metabolic dysfunction has been implicated in PD as well (Cai et al., 2012; Procaccini et al., 2016). Thus, to address the crosstalk between weight loss and PD is quite necessary and metabolic manipulation may provide a promising therapeutic alternative in future management of PD.

Weight loss and malnutrition is the result of a negative energy balance, which means that energy expenditure exceeds intake, resulting in a status of fat loss and malnutrition. Generally speaking, weight loss and malnutrition is commonly observed in neurodegenerative diseases, such as PD, AD, and amyotrophic lateral sclerosis (ALS). Present evidence suggests that inflammation and mitochondrial impairment play an important role in neurodegenerative diseases (Xu et al., 2013; Wei et al., 2016; Wang et al., 2017), which may be also responsible for energy balance (Aziz et al., 2008). In the context of PD, our basic understanding of mechanism of weight loss and malnutrition is limited. Both reduced energy input and increased energy output are postulated as the cause, but neither has been proven.

Accordingly, here in this review, we review current research advances on weight loss and malnutrition in detail in the context of PD, describing the weight and nutritional status, and discussing its correlation with PD and related potential determinants. Also, we discuss the importance of early detection and management of weight loss and malnutrition in PD, which may contribute considerably to overall management and therapy of PD in future clinical settings.

## Aspect 1: weight/malnutrition status in PD

Although James Parkinson reported weight loss in his first publication on PD in 1817, only recently has the attention been focused on body weight change in PD patients. In general, several lines of evidence have shown that PD patients have a lower body weight compared to age matched healthy controls (Beyer et al., 1995; Chen et al., 2003). Patients with PD appear to be four times more likely to experience weight loss than the matched groups (Beyer et al., 1995). The amount of weight loss in PD patients documented in the literature varies from 52 to 65% (Abbott et al., 1992; Beyer et al., 1995), with a mean loss of 3–6 Kg. Although the amount of weight loss tends to be modest in most circumstances, sometimes it can exceed 12.8 Kg (Abbott et al., 1992). A recent meta-analysis on BMI in PD including 871 PD patients and 736 controls suggests that PD patients have a significantly lower BMI than controls (overall effect 1.73, 95% Cl 1.11–2.35, *P* < 0.001) (van der Marck et al., 2012). In line with these data, the MitoPark transgenic mouse model, which was the first to replicate the cardinal clinical features, also exhibited weight loss along with progressive neurodegeneration (Li et al., 2013). The data indicate that progressive loss of body weight begins at 16–20 weeks in the animals. In contrast, some recent literatures reported normal weight and even overweight are also prevalent in PD patients. Barichella et al. (2003a) evaluated the rate of underweight among 364 PD patients at an Italian referral center, of which 134 patients (37%) were overweight, 92 (25%) obese, 127 (35%) normal weight, and only 11 (3%) underweight. These observations are in accordance with a cross-sectional study including 177 PD patients and 177 healthy controls from a tertiary care center in Mexico city (Morales-Briceno et al., 2012), in which overweight and normal weight were more prevalent in the PD group and underweight was almost negligible when compared with controls. In another case-control study aiming to investigate the relationship between PD and BMI changes during the time before the symptoms set in, Ragonese et al. (2008) found that BMI did not change before PD occurs. A very recent 1-year prospective cohort study confirmed that body weight were stable during the disease course (Lindskov et al., 2016). It should be noted that the prevalence of obesity in Italy (Barichella et al., 2003a) and Mexico city (Morales-Briceno et al., 2012) can be high up to 14 and 30%, respectively. It couldn't be excluded that higher body weight at the beginning of disease may compensate for the body weight loss caused by the disease for many years (Barichella et al., 2003a; Morales-Briceno et al., 2012). In addition, the increase in application of dopamine agonists may also affect the picture of weight status (Barichella et al., 2003a). Thus, with the increasing epidemic of overweight and application of modern antiparkinsonian therapy, body weight may also change. Future large sample prospective studies may help address this issue.

By and large, it is well accepted that unintended weight loss down to malnutrition is a common phenomenon in PD. A systemic review showed that the prevalence of malnutrition ranged from 0 to 24% in PD patients, while 3–60% of PD patients were at risk of malnutrition (Sheard et al., 2011). Such variability may be attributed to the use of different assessment tools. For instance, based on the Mini-Nutritional Assessment (MNA), malnutrition rate in PD patients varies from 0 to 25.5% and 19.66 to 34.3% of PD patients are at risk of malnutrition (Fereshtehnejad et al., 2014), while using BMI as a marker, a cut-off value of <20% for under-nutrition identified that over 15% of the study group were in under-nutrition status. Given that most of participants with PD tend to fall in a group of younger, higher functioning with better nutritional status, the malnutrition rate reported in literature is likely to be under-estimated. What is noteworthy is that PD patients with normal weight or even weight gain may also have problem with malnutrition or be at risk of malnutrition. One study showed that although PD patients may have stable body weight, there seems to be redistribution in body composition from muscle to fat, which may suggest PD patients might develop sarcopenia (Lindskov et al., 2016). That is to say, PD patients may suffer from malnutrition even in the absence of weight loss. Accordingly, it is sensible to make a comprehensive assessment on weight and nutrition status in order to early detect potential nutritional risk.

Weight loss and malnutrition are not benign phenomena during the course of PD. PD patients in low BMI group showed lower scores of the K-MMSE and 3MS compared to stable BMI group, implying the potential relation between weight loss and cognitive decline in PD patients (Kim et al., 2012). Low BMI and malnutrition is one such risk factor for osteoporosis in PD patients, which deserves more attention for the concomitant risk of fractures (Schneider et al., 2008; van den Bos et al., 2013; Malochet-Guinamand et al., 2015). In addition, a growing body of evidence suggested weight loss and malnutrition in PD was associated with worsening life qualities (Sheard et al., 2014; Akbar et al., 2015). With respect to survival, only one study explored the association between changes in BMI and survival among persons with PD (Wills et al., 2016). According to this study, changes in BMI was not associated with survival after adjusting for covariates although there was inverse correlation between BMI changes and UPDRS score variations. One thing to note is the low number of death in the study limits the results. Moreover, low body weight patients tend to receive significantly higher daily dose of levodopa per kilogram body weight, which may contribute to developing dyskinesias (Sharma et al., 2006, 2010).

Collectively, although unintended weight loss and malnutrition are well documented in PD patients, normal weight and weight gain are also described particularly in the region where the rate of overweight and obesity is high (Table 1). Considering the global epidemic of overweight and increased application of dopamine agonists and deep brain stimulation (DBS) nowadays, normal weight even obese PD patients may also been frequently observed. Regrettably, we can't identify the person with PD who will experience weight loss or gain. Some researchers reported there exists weight-related phenotypes in PD: phenotype A with a significant olfactory loss and higher initial body weight displaying weight loss as the disease progresses, and phenotype B accompanied with modest loss of olfactory and lower initial body weight and characterized by stable weight or even weight gain during the course of PD (Sharma and Vassallo, 2014). The authors hold the opinion that olfactory impairment in PD may be a marker for the degree and duration of the neurodegenerative progress and may identify PD patients at risk of weight loss. The results should be interpreted cautiously because confounding factors, such as other motor and non-motor symptoms, aren't considered in the study which may be also associated with weight changes in PD. In fact, during the course of PD, nutritional requirement changes as the disease progress, that is, patients may both experience weight loss or gain during the whole course. A prospective study conducted by Chen et al. (2003) showed that PD patients may have weight loss before the onset, weight gain in the first 10 years after diagnosis and weight loss as the disease further progress.

**Table 1:** Summary of literature on weight change in PD patients.

**Reference**	**Study type**	**Subjects**		**Results**
		**Composition**	**PD characteristic**	
Durrieu et al., 1992	Case-control study	65 PD(37 M, 28 F) 68 HC(30 M, 38 F)	Age: M(64.6 ± 1.6) years; *F*(66.4 ± 1.3) years	Women PD patients exhibited a significant weight loss.
Beyer et al., 1995	Case-control study	51 PD 49 HC	Age: (68.4 ± 8) years; Stage: H-YII38; H-YII 12; H-Y IV1	Patients with PD were four times more likely to report weight loss than the matched control subjects.
Chen et al., 2003	Prospective study	47,696 M 117,035 F	_	Weight loss is a continuous process during the follow-up stage.
Lorefalt et al., 2004	Longitudinal studies	2 PD(10 M, 18 F) 28 HC(10 M, 18 F)	15 newly diagnosed; 13 treated with L-dopa 5 ± 2.7 years	Weight loss is common in PD patients especially with serious PD symptoms.
Uc et al., 2006	Longitudinal studies	49 PD(34 M, 15 F) 78 HC(29 M, 49 F)	Age: 57.5 ± 1.6 years	PD patients experienced significant weight loss compared to controls.
Bachmann et al., 2009	Cross-sectional studies	166 PD	-	Advanced PD patients have a reduced BMI compared to the general population.
Sharma and Turton, 2012	Longitudinal studies	99 PD	55 Anosmic (aged 72 ± 8 years); 44 hyposmic	Anosmic group lost weight during the previous 4 years while hyposmic group tended to gain weight.
Vikdahl et al., 2014	Longitudinal studies	58 PD(36 M, 22 F) 24 HC(13 M, 11 F)	H-Y2.0 (2.0;2.5) UPDRS III Subtotal 23.5(18.0; 31.3)	Weight gain and increased central obesity in the early phase of PD.
Lindskov et al., 2016	Longitudinal studies	65 PD(35 M, 30 F)	Age:68.1 ± 8.1 years; PD duration: 6.9 ± 3.2 years	Body weight remained stable during the 1 year follow up.

*M, Male; F, Female; H-Y, Hoehn & Yah; PD, Parkinson's disease; HC, healthy controls; BMI, body mass index*.

## Aspect 2: relationship between weight loss/malnutrition and PD

It has long been recognized that there is a relationship between weight loss and PD. Amounting evidence support weight loss may begin at the early stage of PD, even several years before the diagnosis, and may be more pronounced in patients with greater disease severity (Chen et al., 2003; Lorefalt et al., 2004). A prospective cohort study involving 10,812 men reported that subjects who lost 0.5 units of body mass index per decade during the follow-up time had more than a 2-fold risk for developing PD when compared with men having stable BMI (Logroscino et al., 2007). Given this considerable list of association between weight loss and PD, one is left asking: whether it is a chicken or egg problem?

Evidence from previous studies revealed that weight loss may be linked to pathogenesis of PD. Several studies observed increased plasma concentration of organocholrine pollutants following weight loss (Kiviranta et al., 2005). As organochlorine compounds are lipophilic property and stored in fat in living organisms, the course of weight loss results in high plasma concentration of organochlorine compounds. Subjects, who experience weight loss either by hypocaloric restriction or bariatric surgery, display increased total plasma organocholrine concentration (Teasdale et al., 2007). Since a growing body of evidence has shown a link between exposure to pesticides and the risk of PD, weight loss may contribute to PD pathogenesis due to the increase of plasma organochlorine and pesticide concentration. A recent cohort study involving 398 *de novo* patients with PD found the close relationship between weight loss and nigrostriatal deletion (Lee et al., 2016). The associations between BMI and dopamine transporter (DAT) activity were analyzed by virtue of quantitative analyses of 18F-FP-CIT scanning. After adjustment for confounding factors such as age, gender and disease duration, a multivariate analysis revealed that BMI was significantly associated with DAT activity in all striatal sub-regions. These data indicate that a lower body weight is associated with lower density of nigrostriatal dopamine in PD. In addition, patients experiencing weight loss appeared to have lower plasma leptin and ghrelin levels compared with healthy controls (Evidente et al., 2001; Andrews et al., 2009; Fiszer et al., 2010), both of which play an essential role in energy balance regulation in organism. More recently strong evidence showed that peripheral leptin and ghrelin signals were implicated in pathogenesis of PD as revealed in AD (Weng et al., 2007; Andrews et al., 2009; Bayliss and Andrews, 2013). Leptin was able to protect neuroblastoma and neural dopaminergic cells from injury by 6-hydroxydopamine (6-OHDA) and 1-methy-4-phenylpyridinium (MPP+) possibly via a PI3K/Akt/MEK-dependent pathway (Weng et al., 2007). Likewise, ghrelin provide neuroprotective effects through 5′adenosine monophosphate-activated protein kinase (AMPK) activation and regulate mitochondrial function (Bayliss and Andrews, 2013).

**Figure 1:**
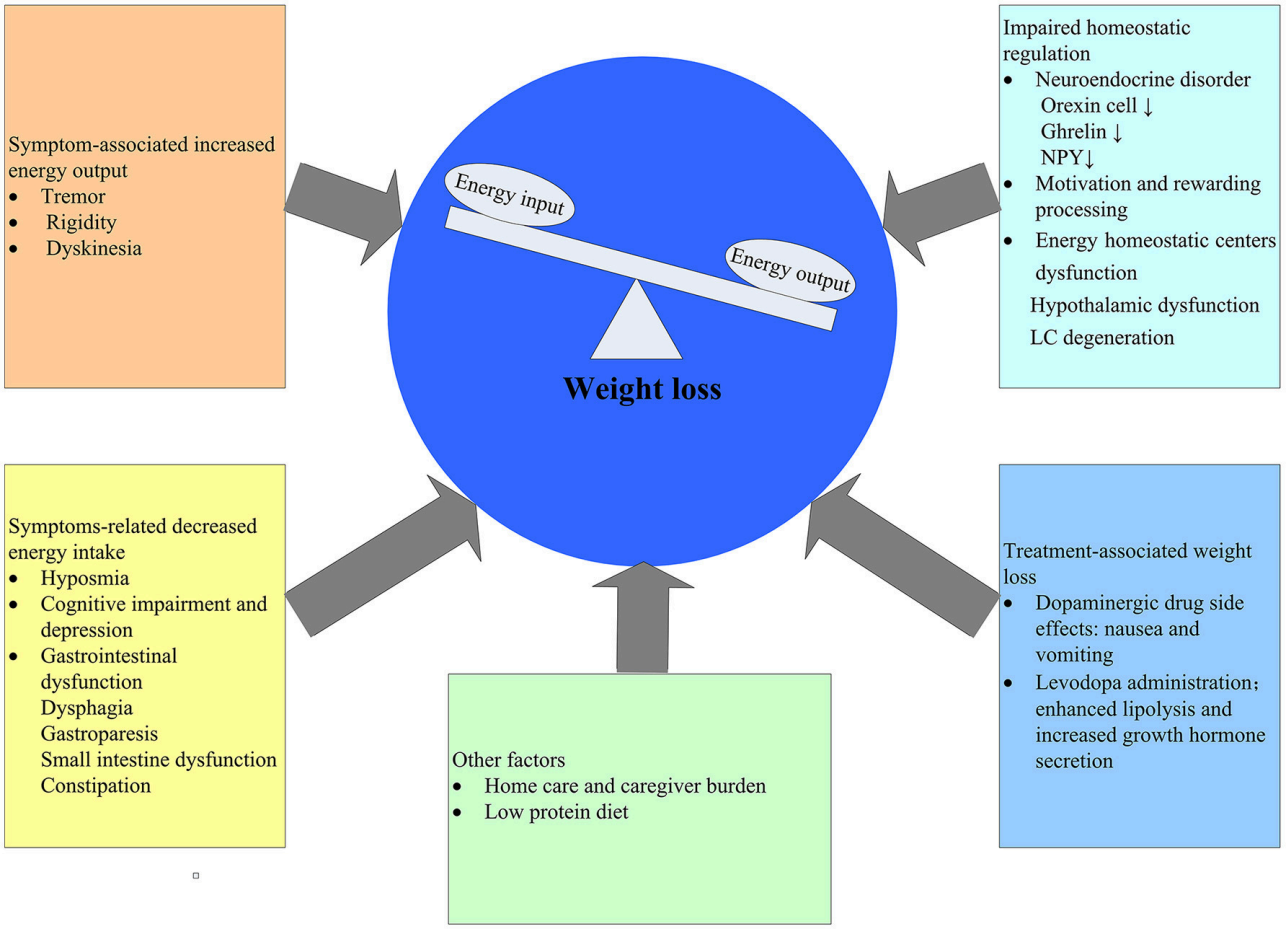
Postulated determinants of weight loss in PD. NPY, NeuropeptideY; LC, locus coreruleus.

Given the lengthy latency period of PD, it cannot be excluded that the observed weight loss before the diagnosis of PD may stem from subclinical effects of PD rather than an independent pathogenesis. That is to say, weight loss is the consequence of PD. There are ample evidences supporting the effect of PD on weight loss for this time being (Figure 1).

Still, in light of these data, the relationship between weight loss and PD has not fully been understood yet. The picture is complex and multi-dimensional in that weight loss may contribute to PD pathogenesis and PD-related motor and no-motor symptoms can influence body morphology. Thus, it is difficult to delineate the relationship between weight loss and PD. With this challenge in mind, longitudinal studies with large sample size and adequate follow-up duration will help to unravel the cause-effect relations. Considering the interaction between weight loss and PD, it is sensible to conduct longitudinal studies at least 5 years before the diagnosis to exclude the effect of PD on weight status. Of note, to address this crosstalk between PD and weight loss will better understand the pathogenesis of PD and may provide a new target to intervene in PD progression. Some studies have observed improvement in nutritional status resulted in life quality improvement (Akbar et al., 2015). As a matter of fact, emerging evidence suggests that there is a strong correlation between metabolic dysfunction and neurodegeneration (Cai et al., 2012; Procaccini et al., 2016). Metabolic manipulation holds great potential to treat neurodegenerative diseases such as AD and PD. Leptin and gherin has been proven to have neuroprotective and disease-modifying effects on PD (Ho et al., 2010; Bayliss and Andrews, 2013). In summary, further studies trying to find out the relationship between weight loss and PD is necessary, and metabolic manipulation as a new promising target should be addressed in future studies.

## Aspect 3: mechanism of weight loss/malnutrition in PD

Body weight and nutritional status regulation is a complicated progress in the body. Through a process known as energy homeostasis, the body maintain body weight. In the context of PD, disease-related and treatment-related factors may affect the energy balance. In this section, we will try to present evidence accounting for weight loss/malnutrition from basic mechanism of energy balance to detailed potential determinants.

## Basic mechanisms

Body weight control results from complex interaction of peripheral signals involving the gastrointestinal tract, adipose tissue and muscle with the central nervous system (Moehlecke et al., 2016). Based on the law of conservation of energy, energy input and energy output need to be regulated in order to maintain neutral energy balance. Energy input is involved in intake, digestion, and absorption of energy from nutrients, while energy output is divided into four types: resting energy expenditure (REE), thermic effect of food, physical activity and adaptive thermogenesis (Ricquier, 2006). Through a process known as energy homeostasis, the body adjust food intake in response to changing energy requirement. In summary, peripheral signals of energy reserves reach neurons in the mediobasal hypothalamic arcuate nucleus and brainstem, where information is integrated and communicated with higher brain regions such as the anterior forebrain mesolimbic reward system. The information, then, is passed on to several other hypothalamic nuclei, including the paraventricular nucleus and the lateral hypothalamic area, finally orchestrating behavioral, endocrine, and autonomic responses to regulate intake and expenditure of energy (Schwartz et al., 2000; Remmers and Delemarre-van de Waal, 2011).

Weight loss and malnutrition is a result of negative energy balance, which means that energy output exceeds energy input. In the early stage of PD, non-motor symptoms, such as sensory dysfunctions (smell, taste), neuropsychiatric symptoms (depression, cognition), gastrointestinal dysfunctions have been linked with weight loss in PD patients due to decreased food intake (Aiello et al., 2015; Fasano et al., 2015). As diseases progress, motor symptoms such as tremor, rigidity and dyskenisas arise, all of which may contribute to increased energy expenditure. However, there are still some problems with this hypothesis. A number of studies have reported although PD patients tend to lose weight in the early stage, the calorie intake is increased (Chen et al., 2003; Lorefalt et al., 2004) as well. One possible explanation is that increased energy expenditure may play a role in the early stage and the increased calorie intake is a compensation for weight loss. As a matter of fact, weight loss in PD is multifactorial involving many aspects (Figure 1). Reduced energy input and/or increased energy output have been postulated as the cause, both of which have not yet been proven. In addition, the possible mechanisms by virtue of which PD could induce weight loss may be different in the various stages of disease. Reasonably, in the early stages of PD increased energy expenditure would be the main cause, whereas in the advanced stages the major determinant of weight loss is likely to be a decreased energy input.

## Postulated determinants of weight loss/malnutrition in PD

### Inadequate energy input in PD patients

#### Hyposmia

Olfactory deficits have been described for many years and can precede the motor onset of PD (Berendse et al., 2001; Ponsen et al., 2004). Studies have shown 80–96% of PD patients have olfactory impairment (Doty et al., 1988; Haehner et al., 2009), and olfactory deficits could distinguish sporadic PD from essential tremor, dystonic tremor and progressive supranuclear palsy (PSP) (Busenbark et al., 1992). Since the sense of smell, an important aspect of food appeal, plays a considerable role in the perception of taste (Landis et al., 2009), it is plausible that olfactory dysfunction may evoke decreased dietary intake and weight loss. In PD patients, the relationship between olfactory impairment and weight loss has been reported, although not consistently (Lorefat et al., 2006; Sharma and Turton, 2012). It was found that low intake of protein was associated with low performance on the olfaction test in PD patients and controls (Aden et al., 2011). However, another study suggested that there was no significant difference between *de novo* and advanced PD patients with respect to olfaction (Palhagen et al., 2005). A recent study did not report any improvement in olfaction in PD patients following unilateral pallidotomy (Ondo et al., 2000), although they experienced significant weight gain. Methodology issues may account for the inconsistent results. For example, inquiries rather than physical tests are used to estimate olfaction dysfunction in some studies and it may lead to bias in the results. In conclusion, some studies may show a link between weight loss and hyposmia in PD patients, nevertheless, the evidence is far from ample. Further studies including a large sample of patients and accurate assessment on olfaction should be conducted and olfaction changes before and after DBS should also be surveyed to address this issue.

## Cognitive impairment and depression

The dementia is more common and severe in the late stages, the prevalence of which is about 20% of PD cases in cross-sectional studies (Munhoz et al., 2010) and 80% in longitudinal studies (Aarsland et al., 2008). Furthermore, mild cognitive impairment may be present early in the course of PD. The characteristics of cognitive impairments in PD are attention, executive dysfunction and visuospatial processing deficits (Emre et al., 2014), which may be implicated in eating behavior. Several studies have indicated that PD patients with impaired cognitive function tend to be at increased risk of weight loss (Lorefalt et al., 2004; Uc et al., 2006; Kim et al., 2012; Sheard et al., 2013b), while another study has not observed this correlation (Fiszer et al., 2010). It is important to note that some studies excluded patients with dementia or cognitive dysfunctions from their sample and this exclusion criterion might sweep the relation between cognitive decline and weight loss under the carpet. Intriguingly, although patients treated with DBS (STN or GPi) may experience weight gain during a follow-up period, subtle cognitive declines has been announced after the surgery by some studies (Witt et al., 2008; Odekerken et al., 2013; Wu et al., 2014; Combs et al., 2015). This cognitive side-effect questions the effect of cognition on weight loss in PD. Thus, further studies are needed to corroborate the relation between cognition and weight loss in PD. Since cognitive deficits progress with the disease, a long observation time would be needed and patients with different severities of cognitive decline should be included.

Apart from cognitive impairment, mood disorder may also affect eating behaviors. Depression is the most common mood disturbance and occurs as early as well as advanced PD with a prevalence around 35% of the patients (Aarsland et al., 2011). It is believed that depression can cause a decrease in appetite and contribute to a cascade of neuroendocrine changes which can disrupt the regulation of eating (Kishi and Elmquist, 2005). In PD patients, the association between depression and weight loss or malnutrition has been observed (Sheard et al., 2013b; Kim et al., 2014), in which Geriatric Depression Scale or Beck Depression Inventory scores were significantly higher in malnutrition PD group. Interestingly, a recent systematical study involving 215 outpatients diagnosed with PD reported that the presence and severity of depression were associated with reduced BMI in male but not in female PD patients (Pilhatsch et al., 2013). The effect of DBS on improvement in depression has also been observed by some studies with a mild improvement in mood after administration of DBS (STN or GPi) (Ardouin et al., 1999; Okun et al., 2009; Wang et al., 2016). In light of these data, evidence for a depression-weight correlation is relatively weak and warrants further studies.

## Gastrointestinal dysfunction

### Dysphagia

Dysphagia, which can occur in all phase of swallowing (oral, pharyngeal, and esophageal), is commonly observed in patients with PD with prevalence ranging from 9 to 77% (Fasano et al., 2015). Although dysphasia is generally considered as a late complication with median latencies of 130 months (Muller et al., 2001), sometimes it can develop early in the course of PD, occasionally even the presenting clinical feature (Thomas and Haigh, 1995; Potulska et al., 2003). Dysphasia has been put forwarded as being responsible for weight loss in PD on the basis of several studies (Nozaki et al., 1999; Kashihara, 2006; Lorefalt et al., 2006; Barichella et al., 2008). Chewing and swallowing disorder, limiting both solids and liquids intake, may be responsible for decreased energy intake (Lorefalt et al., 2006). In addition, swallowing difficulties may result in drooling (Kalf et al., 2007) and consequently increased risk of pneumonia (Nobrega et al., 2008), which may further disturb eating behaviors. In contrast, one study, in which 281 patients with PD and 86 patients with progressive PSP were assessed, indicated that there was no appreciable differences between the two groups with respect to the mean body weight, height, and BMI. Given that patients with PSP have more serious dysphagia than counterparts with PD, the author questioned the role in which dysphagia played in weight loss in PD (Jankovic et al., 1992). Evidence varies across studies as to the effect of DBS on swallowing function in PD. Some researchers suggested DBS significantly improved the swallowing ability of PD patients (Ciucci et al., 2008; Lengerer et al., 2012; Silbergleit et al., 2012), while others reported both STN- and Gpi-DBS were associated with deterioration of swallowing function (Hariz et al., 2008; Robertson et al., 2011; Troche et al., 2013, 2014). Methodological difference may contribute to the inconsistent results. For example, the assessment tools and measures, lead locations (DBS or GPi), number of leads (unilateral or bilateral) vary across studies. Further studies, allowing for the factors above, will be of some help to understand the impact of DBS on swallowing and better illustrate the relationship between dysphagia and body weight in PD.

## Gastroparesis

Gastroparesis is frequently observed in patients with PD and can emerge in both early and advanced stages of PD, whose prevalence ranges from 70 to 100% (Goetze et al., 2006; Heetun and Quigley, 2012). Gastroparesis may give rise to nausea, vomiting, early satiety, excessive fullness, bloating, abdominal discomfort and consequent weight loss secondary to impaired gastric emptying (Edwards et al., 1991; Parkman et al., 2004; Fasano et al., 2015). Since levodopa is absorbed in small intestine, delayed gastric emptying may generate pharmacokinetic implications, interfering with its absorption, and finally causing motor fluctuations in individuals with PD (Djaldetti et al., 1996; Pfeiffer, 2003). In PD patients, although improvement in gastric emptying has been observed in patients receiving DBS, no association so far has been reported between gastric emptying improvement and weight gain after DBS (Arai et al., 2012; Basiago and Binder, 2016). Further studies investigating gastroparesis and weight change before and after DBS are of some help to address the role gastroparesis playing in weight change in PD.

## Small intestine dysfunction

Although small intestine dysfunction have been observed in patients with PD for a long time (Lewitan et al., 1951), very little literature can be available nowadays partly because the assessment methods of small intestine transit time are limited and imprecise (Szarka and Camilleri, 2012). In one study, the small bowel transit time in PD patients, assessed by SPECT after swallowing of a specially prepared capsule containing technetium 99 m, was prolonged in comparison with healthy controls (Dutkiewicz et al., 2015). In consistent with these data, in 1-methyl-4-phenyl-1,2,3,6-tetrahydropyridine (MPTP) rats model, disorder of migrating myoelectric complex activity was reported which could be ameliorated by use of levodopa (Eaker et al., 1987).

Impairment in small intestine motility may be responsible for small intestinal bacterial overgrowth (SIBO), whose prevalence ranges from 54 to 67% in PD patients (Gabrielli et al., 2011; Fasano et al., 2013; Tan et al., 2014). SIBO is a malabsorption syndrome manifesting with bloating, diarrhea, malabsorption, weight loss, and malnutrition. Bacteria in SIBO may not only disrupt mucosal integrity, producing mucosal inflammation and increasing intestinal permeability, but also metabolize intra-luminal nutrients (fructose, lactose, sorbitol, protein) in the small bowel (Nelis et al., 1990; Fasano et al., 2013), all of which are responsible for malabsorption. Along with this phenomenon, deconjugation of bile acids by bacteria may contribute to malabsorption of fat and liposoluble vitamins (Fan and Sellin, 2009). There is also some evidence SIBO may interfere with levodopa absorption and is associated with motor fluctuations (off-time, delayed on-time, and no on-time) (Gabrielli et al., 2011; Fasano et al., 2013, 2015). In PD patients, the association between SIBO and BMI has been surveyed and no difference in BMI was found between groups (SIBO-positive and SIBO-negative) (Gabrielli et al., 2011; Fasano et al., 2013; Tan et al., 2014). Small sample size and the lack of accuracy in assessment of SIBO may misinterpret the results. In conclusion, the recognition of small intestine dysfunction in PD is limited, and the literature on the role of small intestine dysfunction, especially SIBO, in weight loss in PD is scant. Further studies including large sample size and accurate assessment on small intestine dysfunction are needed.

## Constipation

Constipation, defined as fewer than three bowel movements per week, is the most common gastrointestinal symptom in PD, whose prevalence varies between 24.6 and 63% (Stirpe et al., 2016). It may precede the motor dysfunction in PD patients by many years (Abbott et al., 2007; Ross et al., 2012). The pathophysiologic basis for constipation is slowed transit of fecal material through the colon, which results in some symptoms such as bloating, abdominal discomfort, hard stool and straining (Johanson and Kralstein, 2007). In PD patients, one study reported that malnutrition was associated with some non-motor symptoms including constipation, vomiting, loss of interest, inability to concentrate and sadness, of which constipation was one of the most important predictors (Wang G. et al., 2010). In consistency with the results, another study, in which the malnutrition in community dwelling people with PD was investigated, showed that constipation was one of risk factors of malnutrition (Sheard et al., 2013a). However, a more recent study suggested that the number of dysautonomia symptoms (dysphagia, sialorrhea, and constipation) rather than single symptoms were associated nutrition risk (Barichella et al., 2013). Although some studies have observed constipation was improved after STN-DBS (Zibetti et al., 2007; Chou et al., 2013; Jafari et al., 2016; Krygowska-Wajs et al., 2016), no study available investigated the relationship between weight change and constipation improvement after DBS. In summary, constipation seems to play a role in malnutrition although the results remain obscure. Further large prospective studies investigating the relation between weight loss and constipation is of some help, and it is also necessary to assess the body weight and constipation before and after DBS surgery to illustrate the effect of constipation on weight.

## Increased energy output

There are some evidences that weight loss in PD patients may be attributed to increased energy expenditure (Broussolle et al., 1991; Chen et al., 2003). By using indirect calorimetry methods, Broussolle et al. (1991) suggested that energy expenditure in PD patients was higher as compared to healthy groups and essential tremor groups. This phenomenon was also observed in α-synuclein A53T mutant mice (Rothman et al., 2014). A53T mice displayed greater energy expenditure, measured by a Comprehensive Lab Animal Monitoring System, and were resistant to HCD-induced obesity, implying that α-synuclein pathology was responsible for metabolic abnormalities in mouse models. However, given that energy expenditure consists of four components, these studies failed to point out which part of energy expenditure played a role. Although in A53T mutant mouse model α-synuclein pathology may be considered as a cause of metabolic impairment, this result has not been confirmed in human subjects.

In contrast, Toth et al. (1997) reported that daily energy expenditure, measured by double labeled water, was lower in PD patients partly owing to reduced physical activity. This discrepancy may be explained for differences in disease duration, clinical stages and medication state across studies.

## Increased resting energy expenditure

Using indirect calorimetric methods, Levi et al. and Markus et al. consistently observed increased REE, which was significantly associated with muscle rigidity, both in the untreated state (off state) and treated state (on state) (Levi et al., 1990; Markus et al., 1992). In consistency with the results, Marianna et al also found that REE was higher in the off state, and could be decreased by 8% after dopaminergic therapy (Capecci et al., 2013). In contrast, Delikanaki-Skaribas et al. (2009) and Toth et al. (1997) reported that there was no difference in REE between neither PD patients and healthy controls nor weight loss and weight stable PD patients. Moreover, according to recent data, normalization of REE may contribute to the weight gain after DBS surgery (Macia et al., 2004; Perlemoine et al., 2005; Montaurier et al., 2007). However, other authors reported that REE remains unchanged in PD patients treated with STN-DBS (Lammers et al., 2014).

## Tremor and rigidity

Tremor is a common clinical characteristic of PD with a prevalence of 75% in PD patients. It may manifest at the onset and increase in amplitude as the disease progress in some patients (Rodriguez-Oroz et al., 2009), which may increase energy expenditure. Although some researchers posited tremor as factors of weight loss in PD patients, few prospective studies investigated the relationship between tremor and weight loss. A retrospective studies reviewing patients undergoing DBS found that patients gained weight because of decreased energy expenditure due to subsidence of chronic tremor (Tuite et al., 2005).

Apart from tremor, rigidity may be another factor affecting REE during off state. Rigidity in PD is characterized as increased muscular tone to palpation at rest, reduced distension to passive movement, increased resistance to stretching and facilitation of the shorten reaction (Andrews et al., 1972), involving in both flexor and extensor. Association between rigidity and increased REE has been put forwarded by several studies (Levi et al., 1990; Markus et al., 1993), while few studies focused on the relationship between rigidity and weight loss in PD patients. Moreover, reduced muscle stiffness may play a role in weight gain after DBS surgery in patients with PD. In a study, 24 patients with PD were placed in a calorimetric chamber for 24 h before and after DBS surgery, in which body composition and energy expenditure were measured (Montaurier et al., 2007). Reduced REE and weight gain were observed after surgery while energy intake remained unchanged. The authors considered lowered energy requirements after surgery as post-operative weight gain, which was partly attributed to reduced muscle stiffness and motor fluctuations. In conclusion, studies on association between tremor, rigidity and body weight in PD patients are rare and some studies may exclude patients with serious tremor or rigidity. Further prospective study with longer duration investigating the relation between tremor, rigidity and body weight change in PD patients should be conducted.

## Dyskinesia

Although levodopa is still the most effective drug for treating motor symptoms of PD, long term treatment is accompanied with dyskinesa, partly due to pulsatile stimulation of striatal postsynaptic receptors. Long term follow-up studies showed the incidence of levodopa-induced dyskinesa was 40–50% at 5 years, 52–78% at 10 years, and 94% after 15 years (Zesiewicz et al., 2007). When dyskenisa occurs, irregular fast and slow movements of facial muscles, jaw, tongue, neck and trunk are seen in patients with PD, which may increase energy expenditure. An anthropometric study reported moderate or serve dyskinesia as the most strongly related clinical parameter to undernutrition, in which anthropometric indices were assessed and correlated with clinical parameter (Markus et al., 1993). In consistent with the results, one survey involving mainly patients with fluctuations and dyskenisas showed that advanced PD patients had a lower BMI as compared to an age matched group, which could be partly related to increased daily time with dyskenisas (Bachmann et al., 2009). Moreover, mounting evidence suggested that both DBS of STN and GPI were effective to reduce dyskinesia (Anderson et al., 2005; Odekerken et al., 2013). In addition, there are some evidence showing that reduced dyskenisa was related to weight gain after DBS surgery (Macia et al., 2004; Montaurier et al., 2007). In contrast, PD patients undergoing bilateral STN-DBS displayed an average weight gain of 13% within 16.3 ± 7.6 months in all seven patients with PD, which was not associated with changes in post-surgery dyskenisa scores (Moro et al., 1999). This is consistent with another two follow-up investigations on weight gain after unilateral pallidotomy (Ondo et al., 2000; Vitek et al., 2003). In summary, literature available at present does not reveal the relationship between dyskenisa and body weight change in PD, and further studies need to be conducted to address this issue.

## Physical activity

Akinesia and bradykinesia are common motor manifestations due to loss of dopamine in the posterior putamen (Rodriguez-Oroz et al., 2009), both of which can limit patients' daily activities. In addition, fatigue, affecting 50% PD patients, is another frequently disabling aspects (Friedman and Friedman, 2001), increasing patients' period of recumbent rest. Regrettably, there exist few literatures on association between physical activity and weight loss in PD, partly owing to the lack of assessment methods. In one study, weight loss was attributed to female gender, age and low physical activity by multiple regression analyses (Lorefalt et al., 2004). In another study, physical activity energy expenditure was higher in weight loss group than weight stable group only by wrist activity monitors and this was not association with weight loss (Delikanaki-Skaribas et al., 2009). However, the assessment methods used in the studies were activity questionnaires and activities monitor which were lack of accuracy. With respect to DBS, only two studies observed physical activity energy expenditure before and after surgery (Barichella et al., 2003b; Jorgensen et al., 2012), both of which found physical activity remained unchanged. However, the authors admitted that the scale used to assess physical activity may be with a low sensitivity (Jorgensen et al., 2012). Lack of appropriate assessment methods may limit the premise measurement of physical activity. Luckily, nowadays wearable technology with robust accuracy validity metrics for free-living monitoring is emerging and should be of some help in the future (Del Din et al., 2016).

## Thermic effect of food and adaptive thermogenesis

Thermic effect of food, defined as the increase in energy expenditure in response to food intake, accounts for 10% of total daily energy expenditure according to previous studies (Poehlman, 1989; Asahara and Yamasaki, 2016). For this time being, no studies ever investigate this aspect in PD patients. There are some evidence indicating that PD patients change eating habits with a higher intake of carbohydrate and a decreased intake of protein compared to controls (Wolz et al., 2009; Aden et al., 2011; Vikdahl et al., 2014). Given that an alteration of food preference, it is reasonable to deduce changed thermic effect of food in patients with PD. However, thermic effect of food and adaptive thermogenesis in PD have not been surveyed yet. Accordingly, future studies on thermic effect of food and adaptive thermogenesis are needed to be conducted to better understood energy expenditure in weight loss in PD.

## Impaired homeostatic regulation

As shown in basic mechanism of energy balance, homeostatic regulation depends on orchestrated interaction of peripheral signals with central pathway. Peripheral signals, mainly neuroendocrine signals, pass on orexigenic, and anorexigenic signals to central nervous system. Central pathway, including motivation and reward processing and energy homeostatic centers, integrate information to regulate appetite and food intake. Dysfunctions in either section may lead to disturbed homeostatic regulation.

## Neuroendocrine disorder

Hypothalamus receives and integrates orexigenic and anorexigenic signals to regulate appetite and food intake. Ghrelin is a powerful orexigenic peptide functioning as increasing food intake and promoting adipogenesis (Steiger, 2004). NeuropeptideY (NPY) is also a strong orexigenic peptide, whose synthesis and release is promoted by ghrein and inhibited by leptin (Li et al., 2016). In the context of PD, an increasing loss of hypocretin (orexin) cell with disease progression is reported (Drouot et al., 2011). In addition, serum levels of orexigenic signals such as ghrelin and NPY are low and paradoxically correlated with BMI (Fiszer et al., 2010). Accordingly, both changes in peripheral and hypothalamus orexigenic signaling may contribute to weight loss in PD.

## Motivation and reward processing

Midbrain dopamine neurons especially the ventral tegmental area (VTA), which project to nucleus accumbens (ventral stratum), limbic systems, and the prefrontal cortex, has been implicated in motivation and reward processing (Luo and Huang, 2016). Therefore, it is reasonable to postulate alternations in food motivation and reward processing in patients with PD, which is characterized by loss of dopamine neurons in the substantia nigra par compacta with relatively spared dopamine neurons in VTA. However, there were no differences with respect to pleasantness rating, which was regard as a good indicator of the reward system function, between PD patients and control groups (Sienkiewicz-Jarosz et al., 2005, 2013). In addition, dopamine depletion in mice has proved no effect on sucrose rewards (Cannon and Palmiter, 2003). One rational explanation is a second compensatory mechanism, such as cerebellar pathway activation, may play a crucial role when dopamine system is out of order (Goerendt et al., 2004). In fact, PD patients showed a reduction in appetitive motivational arousal to appetitive image compared to healthy controls (Shore et al., 2011). In line with this, manipulation of DA neurons leading to reduced dopamine-mediated function inhibited the reward-seeking behaviors, for example making effort to get access to food, although the pleasure associated with rewards did not change (Berridge and Robinson, 1998; Salamone and Correa, 2002). Unfortunately, there are no prospective studies investigating the correlation between altered motivating and reward and body weight change in PD patients to date. On the other hand, body weight gain after STN-DBS has been ascribed to changes in the motivation according to some studies (Serranova et al., 2011, 2013). In light of these data, motivation and reward system especially incentive salience may change in PD patients compared to healthy controls, which probably affects their eating behaviors.

## Energy homeostatic centers dysfunction

Defects in energy homeostatic centers in the brain, particularly the hypothalamus and autonomic centers, have been linked to weight loss in PD patients. Postmortem studies suggested a mild to moderate reduction in noradrenaline (NA), serotonin (5-HT), and dopamine (DA) levels in the hypothalamus of patients with PD (Javoy-Agid et al., 1984; Shannak et al., 1994). In line with postmortem observations, functional imaging studies showed that both presynaptic function of monoaminergic terminals and postsynaptic receptor function were reported in disorder, with reduced monoamine storage capacity (Moore et al., 2008) and loss of dopamine D2 receptor binding in PD hypothalamus (Politis et al., 2008). In addition, lewybody pathology, the main pathological hallmark of PD, is reported in the hypothalamic nuclei (Langston and Forno, 1978). More recently, clinicopathological correlation for non-motor systems (NMS), such as weight loss and autonomic symptoms, were performed and patients with and without severe NMS showed no significant difference in severity of hypothalamic a-synuclein pathology. These data indicated that hypothalamic functional deficits rather than lewy body pathology may be responsible for NMS (De Pablo-Fernandez et al., 2016). DBS surgery sheds light on the hypothalamic disorder implicated in body weight change. Mounting evidences have suggested that PD is linked to disorder in energy metabolism which may be normalized after DBS-STN (Montaurier et al., 2007; Strowd et al., 2010). As hypothalamus is very close to the medial limbic tip of the STN, a current diffusion impact on hypothalamus is considered by some researchers as one possible factor in weight gain after DBS-STN (Sauleau et al., 2009), although not consistently (Butson et al., 2006).

Apart from hypothalamus, the locus coreruleus (LC) is also thought to be an important homeostatic control center (Wang Y. et al., 2010), which interacts with hypothalamus via afferent and efferent fibers. LC degeneration has been observed in human postmortem studies (Zarow et al., 2003). The effect of LC degeneration on weight change in PD was corroborated in the 6-OHDA rat model (Guimaraes et al., 2013). According to the study, weight loss was observed only in rat with lesion of the LC and striatum compared to lesion of striatum alone, while chronic DBS-STN abolished the weight variation.

## Treatment-associated weight loss

Nausea and vomiting are common dopaminergic adverse events partly owing to the hyperstimulation on dopamine receptors in the gut and in the area postrema (Perez-Lloret and Rascol, 2010). Almost all the active dopamine-mimetic medications, such as levodopa (Koller, 2000), dopamine receptor agonists (Stowe et al., 2008), and catecholomethyl transferase enzymes (Brooks, 2004) may give rise to nausea and vomiting limiting energy intake. However, these adverse reactions are usually at the onset of therapy and become tolerated over time.

In addition, several studies suggested that long-term levodopa administration may result in enhanced lipolysis secondary to hyperinsulinaemia and increased growth hormone secretion causing reduction of fat and weight loss in PD patients (Vardi et al., 1976; Hanew and Utsumi, 2002). Notably, the observations above using levodopa may have limited clinical significance because levodopa is usually prescribed in combination with a decarboxylase inhibitor. In contrast, another study found the contrary results by assessing levodopa/benserazide on metabolic responses in PD patients (Adams et al., 2008). The authors observed a switch from lipid to carbohydrate in both adipose tissue and skeletal muscle after assessing, indicating no influences of levodopa/benserazide on lipolysis.

## Other factors

### Home care and caregiver burden

Patients with PD are tend to lose independence as disease progress and require more and more assistance in everyday life (Bjornestad et al., 2016). They usually need some help for managing daily living and dietary intake, such as shopping, cooking and eating. A study demonstrated that living alone was one of significant predictors of malnutrition (Andersson and Sidenvall, 2001), implying the potential role of health care in body weight in PD. On the other hand, patients care is burdensome and time investment, which have negative impact on the caregivers' health status (Schrag et al., 2006; Martinez-Martin et al., 2008). In AD, caregiver burden has been considered as an independent risk of factors for weight loss even in a short-term (3 months) (Gillette-Guyonnet et al., 2000; Grun et al., 2016). To date, there are no studies investigating the correlation between caregiver burden and weight loss in patients with PD.

## Low protein diet

Amino acids compete with levodopa for transportation in the small intestine and across the blood brain barrier (Cotzias et al., 1967). Accordingly, low-protein diets (<0.8 g/kg of ideal weight/day) are proposed to patients with motor fluctuations, which on average proves to be effective to improve motor function (Cereda et al., 2010). However, such diets may cause weight loss and worsen the nutrition status of the patients with PD (Riley and Lang, 1988; Barichella et al., 2006; Virmani et al., 2016). Although the weight loss induced by low-protein diet was usually mild, sometimes it may be severe (>5% of body weight) (Riley and Lang, 1988).

## Aspet4: evaluation and management

The assessment measures available at present include biochemical indicators (serum albumin, prealbumin, total lymphocyte count), anthropometric measurement (BMI, TSF, and AMC), changes in weight, nutrition screening and assessment tools (MNA, ANSI, SGA, and MUST) (Sheard et al., 2011; Table 2). Biochemical indicators are commonly used in the clinic and are sensitive for the early diagnosis of acute undernutrition. However, laboratory biochemical tests are susceptible to be affected by external factors and tend to be nonspecific for the diagnosis of malnutrition. For instance, renal and/or hepatic insufficiency may have effect on levels of serum albumin and prealbumin. Anthropometric measures and change in weight are convenient, inexpensive and noninvasive. However, as mentioned in aspect1, with the global epedimic of overweight and obesity, using anthropometric parameters such as BMI alone may not be sensitive enough to identify people with risk of undernutrition. Accordingly, nutrition assessment tools are needed to systematically assess nutritional status, which incorporate anthropometry, weight loss, and dietary intake. MNA is a comprehensive geriatric assessment instrument designed to evaluate early malnutrition risk. It is easy to be carried out and takes <15 min (Vellas et al., 1999). Several studies have suggested MNA is a valid and reliable tool to evaluate nutritional status in patients with PD (Barichella et al., 2008; Ghazi et al., 2015). PSGA and MUST are also used by some researchers to assess nutritional status in PD (Jaafar et al., 2010; Sheard et al., 2013a). In the future, further studies involving large-sample, multicenter and multiregion should be completed to corroborate the validation of these assessment tools in PD.

**Table 2:** Nutritional assessment measures available at present.

**Methods**	**Definition**	**Parameters**	**References**
**BIOCHEMICAL INDICATORS**			
Albumin	Visceral protein reflecting the net result of hepatic synthesis, plasma distribution, and protein loss	Albumin <35 g/l as a sign of malnutrition	Omran and Morley, 2000
Prealbumin	A Transport protein for thyroxine	<2 g/l as a sign of malnutrition	Omran and Morley, 2000
TLC	Calculated by multiplying the white blood count by the automated percent lymphocytes	<1,500 per cubic millimeter as a sign of malnutrition	Omran and Morley, 2000
**ANTHROPOMETRIC MEASUREMENT**			
BMI	Weight [kg]/[height in m]^2^	Patients <65 years: BMI <20 risk of malnutrition; Patients>65 years: BMI <21 risk of malnutrition	Markus et al., 1993; Beyer et al., 1995
TSF	Measured in mm at the mid-point between the olecranon and acromion process of the right arm using Harpenden calipers	—	Durrieu et al., 1992
MAC	Measured in cm at the mid-point between the olecranon and acromion process of the right arm	—	Durrieu et al., 1992
AMC	MAC -(TSF^*^0.314)	—	Durrieu et al., 1992
**CHANGE IN WEIGHT**			
Weight loss	Clinically significant weight loss	Loss exceeding 5% in 3 months, or 10% in 6 months	Uc et al., 2006
**NUTRITION SCREENING AND ASSESSMENT TOOLS**			
MNA	A multidimensional instrument including anthropometry, diet, and global assessment	Normal nutritional status (>24) At risk of undernutrition (17–23.5) Malnourished (<17)	Laudisio et al., 2014; Ghazi et al., 2015
MUST	A comprehensive assessment including BMI, weight change, food intake	Low risk (0) Medium risk (1) High risk (≥2)	Jaafar et al., 2010
SGA	A medical history and a physical examination of fat stores, muscle status and fluid status	Well nourished (A) Moderately malnourished (B) Severely malnourished (C)	Sheard et al., 2013a
ANSI	The Australian Nutrition Screening Initiative consisting of 12 questions	No nutritional risk (0) Low nutritional risk (1–3) Moderate nutritional risk (4–5)	Koritsas and Iacono, 2015
		High nutritional risk (>6)	

*TSF, triceps skinfold thickness; MAC, mid-armcircumference; AMC, arm-muscle circumference; MNA, Mini-Nutritional Assessment; MUST, Malnutrition Universal Screening Tool; SGA, Subjective Global Assessment; ANSI, Australian Nutrition Screening Initiative; TLC, total lymphocyte count*.

**Figure 2:**
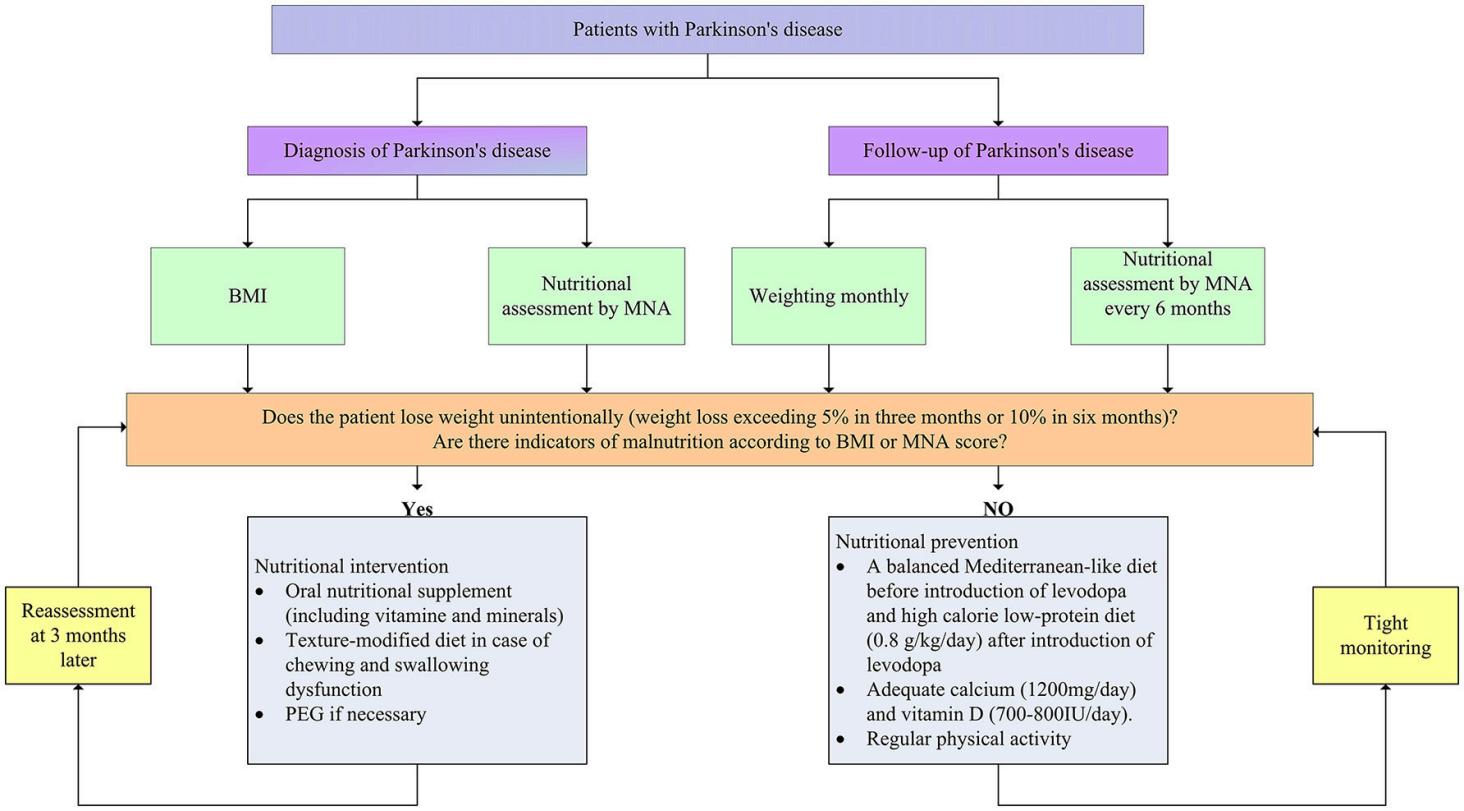
Nutritional assessment and management in patients with Parkinson's disease. BMI, body mass index; MNA, Mini-Nutritional Assessment; PEG, percutaneous endoscopic gastrostomy.

Given that the continuous progress of weight loss and undernutrition, it is of crucial importance to evaluate body weight and nutritional status for every PD patients routinely at the time of diagnosis and follow-up stage in order to ensure early detection and improved outcomes (Figure 2). Clinicians should acquire a comprehensive body weight and nutritional information at the time of diagnosis. BMI and nutritional assessment tools can offer an overview of weight and nutritional status of newly diagnosed PD patients. As weight loss progresses with disease stages, it is necessary to monitor weight and nutritional status routinely. Weighting monthly was advanced by some researchers during the follow-up stage. Moreover, it is necessary to use nutrition assessment tools such as MNA to monitor nutritional status every 6 months throughout the follow-up stage.

Once the weight and nutritional status have been established, early clinical intervention is critical. To date, there is no available guideline for the intervention and prevention of weight loss in PD. However, at least some advice can be advanced according to existing literatures. If patients experience significant weight loss (5% in 3–6 months) or nutritional assessment tools identify patients who have or at risk of malnutrition, intervention measures should be on the way. Recent studies have demonstrated that oral nutritional supplement (ONS) can improve nutritional status or even increase body weight for malnourished groups due to normal aging or neruodegenerative diseases, such as AD and ALS (Salas-Salvado et al., 2005; Gillette Guyonnet et al., 2007). Likewise, a number of studies suggested that ONS could have positive effects on nutritional status and improve quality of life in patients with PD (Sheard et al., 2014). Thus, energy-rich oral nutritional supplements including vitamin and mineral can be suitable for patients with (risk of) malnutrition. However, the drug-nutrient interaction during the supplement of protein has to be considered previously. It is a good choice to counsel nutritionists and physicians to make comprehensive nutritional interventions according to nutritional assessment. Moreover, texture-modified diet is also of some help in case that patients have problem with chewing and swallowing (Logemann, 2007). In addition, at the advanced stage, when appropriate nutrition can't be maintained by other treatments, percutaneous endoscopic gastrostomy may be needed. Nutritional intervention should be conducted in supervision of the caregivers and reassessments are advised to be made 3 months after the nutritional intervention, as it is well accepted that it takes at least 3 months to improve nutritional status (van Asselt et al., 2012).

With regard to patients with normal body weight and nutritional status, measures should be taken to prevent patients against weight loss and (risk of) malnutrition (Figure 2). Firstly, supplement with adequate dietary fiber (30–35 g daily) and fluid intake (1,500 ml daily) is necessary to prevent and reduce constipation (Johanson, 2007). Secondly, before the introduction of levodopa, a balanced Mediterranean-like diet is advocated to prevent weight loss (Sofi et al., 2008). Mediterranean-type diet is characterized by high intake of vegetables, legumes, fruits, and cereals, a high intake of unsaturated fatty acids, but low intake of saturated fatty acids, a moderately high intake of fish, a low-to-moderate intake of dairy products, a low intake of meat and poultry, and a regular but moderate consumption of alcohol. After the application of levodopa, protein-redistribution diet could be helpful for the utilization rate of the drug. As disease progress, low-protein diet (0.8 g/kg/day) is advised to reduce motor fluctuations. It worth noting that calorie intake should be increased to prevent malnutrition in this phage. Thirdly, diet should encompass adequate calcium (1,200 mg/day) and vitamin D (700–800 IU/day) to prevent osteoporosis. Last but not least, physical activity could be proposed to patients with PD which can increase bowel movements (Johanson, 2007), prevent muscle wasting, and stimulate appetite (Gillette Guyonnet et al., 2007). However, there is a paucity of literature regarding optimal exercise intervention for patients at different stages of PD.

## Concluding remarks and future perspectives

Increasing evidence has suggested weight loss is commonly observed in patients with PD. Notably, with the global epidemic of obesity and increased application of modern therapeutic measures such as dopamine agonists and DBS, normal weight even overweight may be also frequently reported in PD patients nowadays. An early identification of patients at risk of weight loss might be of some help to develop measures to prevent weight loss. Recently, only two groups of researchers have assessed profile of weight changes in PD patients among PD subtypes (Sharma and Turton, 2012; Mun et al., 2016). According to the studies, the anosmic group and non-tremor dominant subtypes are associated with weight loss in PD among the different phenotypes respectively. Hence, early detection of olfaction impairment may be predictions for weight loss in the early phase of PD although further corroboration is needed in the future.

Weight loss is not an independent pathogenesis but seems to be coupled with PD pathogenesis as demonstrated in AD (Joly-Amado et al., 2016). In AD, weight loss is one of the criteria for the clinical diagnosis of dementia. Likewise, in the context of PD, weight loss may precede the motor symptoms and be considered as an index for disease progress. To address the crosstalk between weight loss and PD is quite necessary and metabolic manipulation may provide a therapeutic alternative in the treatment of PD in the future.

The pathogenesis of weight loss in PD has not been fully understood. Deceased energy input and/or increased energy output have been posited as possible determinants, but neither has been proven. It seems that energy requirement is changing over time during the course of PD. Future follow-up energy balance studies from early stage to advanced stage might enable understanding of underlying mechanism of weight loss/malnutrition in PD.

Early detection of weight loss and (risk of) malnutrition is critical. The clinicians should monitor weight and nutritional status right from diagnosis and throughout the follow-up period. Regrettably, nutritional assessment measures used nowadays differ across studies. Establishing a consensus standard for nutritional assessment in PD patients should be undertaken by clinicians and nutritionists. The present data on interventions for patients experiencing weight loss and nutritional problems provide only weak evidence for treatment recommendations. Currently, a growing body of evidence suggested improving nutritional status could improve quality of life in PD patients (Sharma and Vassallo, 2014; Wills et al., 2016). However, there are no consistent conclusions on when and how nutritional intervention should be conducted. Future studies focusing on these issues should be on the way.

## Author contributions

Conception or design of the work: TW; Collection of materials: KM, YS, LL, CH, GZ, LW, SG, XG, YX, and FW; Drafting the work: KM, NX, YS, LL, CH, LW, SG, XG, YX, and FW; Review and critique: NX, JH, YS, ZL, and TW; The manuscript has been read and approved by all co-authors.

### Conflict of interest statement

The authors declare that the research was conducted in the absence of any commercial or financial relationships that could be construed as a potential conflict of interest.

## Abbreviations

PD: Parkinson's Disease
AD: Alzheimer's disease
BMI: Body mass index
ALS: Amyotrophic lateral sclerosis
MNA: Mini-Nutritional Assessment
DBS: Deep brain stimulation
DAT: Dopamine transporter
6-OHDA: 6-hydroxydopamine
MPP+: 1-methy-4-phenylpyridinium
AMPK: 5′adenosine monophosphate-activated protein kinase
REE: Resting energy expenditure
PSP: Progressive supranuclear palsy
MPTP: 1-methyl-4-phenyl-1,2,3,6-tetrahydropyridine
SIBO: Small intestinal bacterial overgrowth
NPY: Neuropeptide Y
VTA: Ventral tegmental area
NA: Noradrenaline
5-HT: 5-hydroxytryptamine
DA: Dopamine
NMS: Non-motor systems
LC: Locus coreruleus
ONS: Oral nutritional supplement.
